# Artificial Intelligence Prediction of Cardiovascular Events Using Opportunistic Epicardial Adipose Tissue Assessments From Computed Tomography Calcium Score

**DOI:** 10.1016/j.jacadv.2024.101188

**Published:** 2024-08-28

**Authors:** Tao Hu, Joshua Freeze, Prerna Singh, Justin Kim, Yingnan Song, Hao Wu, Juhwan Lee, Sadeer Al-Kindi, Sanjay Rajagopalan, David L. Wilson, Ammar Hoori

**Affiliations:** a Department of Biomedical Engineering, Case Western Reserve University, Cleveland, Ohio, USA; b Center for Computational and Precision Health, DeBakey Heart and Vascular Center, Houston Methodist, Houston, Texas, USA; c Harrington Heart and Vascular Institute, University Hospitals Cleveland Medical Center, Cleveland, Ohio, USA; d School of Medicine, Case Western Reserve University, Cleveland, Ohio, USA; e Department of Radiology, Case Western Reserve University, Cleveland, Ohio, USA

**Keywords:** Cox, CT calcium score, epicardial adipose tissue, machine learning, major adverse cardiovascular event, radiomics, risk prediction

## Abstract

**Background:**

Recent studies have used basic epicardial adipose tissue (EAT) assessments (eg, volume and mean Hounsfield unit [HU]) to predict risk of atherosclerosis-related, major adverse cardiovascular events (MACEs).

**Objectives:**

The purpose of this study was to create novel, hand-crafted EAT features, “fat-omics,” to capture the pathophysiology of EAT and improve MACE prediction.

**Methods:**

We studied a cohort of 400 patients with low-dose cardiac computed tomography calcium score examinations. We purposefully used a MACE-enriched cohort (56% event rate) for feature engineering purposes. We divided the cohort into training/testing sets (80%/20%). We segmented EAT using a previously validated, deep-learning method with optional manual correction. We extracted 148 initial EAT features (eg, morphologic, spatial, and HU), dubbed fat-omics, and used Cox elastic-net for feature reduction and prediction of MACE. Bootstrap validation gave CIs.

**Results:**

Traditional EAT features gave marginal prediction (EAT-volume/EAT-mean-HU/BMI gave C-indices 0.53/0.55/0.57, respectively). Significant improvement was obtained with the 15-feature fat-omics model (C-index = 0.69, test set). High-risk features included the volume-of-voxels-having-elevated-HU-[-50,-30-HU] and HU-negative-skewness, both of which assess high HU values in EAT, a property implicated in fat inflammation. Other high-risk features include kurtosis-of-EAT-thickness, reflecting the heterogeneity of thicknesses, and EAT-volume-in-the-top-25%-of-the-heart, emphasizing adipose near the proximal coronary arteries. Kaplan-Meyer plots of Cox-identified, high- and low-risk patients were well separated with the median of the fat-omics risk, with the high-risk group having an HR 2.4 times that of the low-risk group (*P* < 0.001).

**Conclusions:**

Preliminary findings indicate an opportunity to use finely tuned, explainable assessments on EAT for improved cardiovascular risk prediction.

Cardiovascular disease is a major cause of morbidity and mortality worldwide,^1^ leading to 17.9 million deaths globally each year.^2^ Numerous risk score methodologies have been developed to predict risks from cardiovascular disease, but these methods often lack sufficient discrimination.^3^ Accurate explainable risk prediction models will provide useful information to patients and physicians for more personalized medications and interventions.

Previous studies have determined the usefulness of coronary calcification Agatston score as obtained from computed tomography calcium score (CTCS) images for cardiovascular risk prediction. The whole-heart Agatston calcium score is one of the most significant single predictors for MACE from noninvasive measurements,^4^, ^5^, ^6^ outperforming clinical factors such as smoking, blood pressure, and total cholesterol.^7^ However, in the early stages of coronary artery disease (CAD), calcification maybe minimal or even nonexistent. Moreover, CAD sometimes involves vulnerable plaque formation without a heavily calcified component to be captured in the Agatston score. One report indicates that half of patients younger than 40 years of age with obstructive CAD on coronary computed tomography angiography have zero Agatston, highlighting the age-dependency of the prognostic value of Agatston.^8^ These findings suggest that there might be room for improvement.

The epicardial adipose tissue (EAT) has garnered significant attention for MACE prediction. Of particular interest is the pericoronary adipose tissue (PCAT), a subset of EAT located adjacent to the coronary arteries. Several studies indicated that PCAT volume and elevated fat attenuation index were associated with coronary inflammation and subsequent MACE.^9^, ^10^, ^11^ Quantitative features obtained from PCAT and EAT have been linked with cardiovascular risk, but this has not been widely examined for prediction of MACE.

There is a pathophysiological rationale for the role of EAT in MACE risk. EAT is in vascular communication with the myocardium and coronary arteries.^12^^,^^13^ As a result, inflammation in EAT can affect atherosclerosis development due to the secretion of proinflammatory and profibrotic cytokines.^14^ It has been determined that inflammation of fat results in higher Hounsfield unit (HU) values.^10^^,^^11^ Notably, EAT is not uniformly distributed and has regional differences. The idea that EAT and PCAT can signal atherosclerosis development has been dubbed the “outside-in theory” of atherosclerosis.^15^ Regarding EAT specifically, current studies have focused on simple features, including EAT volume, mean HU level, and max thickness,^16^, ^17^, ^18^, ^19^, ^20^, ^21^, ^22^ potentially leaving room to identify other important characteristics of EAT. Table 1 reviews published reports of MACE and CAD prediction from EAT features.

**Table 1:** Association of Epicardial Adipose Tissue With MACE or CAD in Previous Publications

First Author, Year	Modality	Cohort	Predictor	Outcome	Results
Cheng et al, 2010^29^	CT	232	EAT volume >125 ${\text{c}\text{m}}^{3}$	MACE	OR: 1.74 (95% CI: 1.03-2.95)
Yang et al, 2023^30^	CT	290	EAT volume >108.3 ${\text{c}\text{m}}^{3}$	MACE	HR: 3.3 (95% CI: 2.1-5.2)
Eisenberg et al, 2020^22^	CT	2,068	EAT volume Mean HU	MACE	HR: 1.35 (95% CI: 1.07-1.68) HR: 0.83 (95% CI: 0.72-0.96)
Goeller et al, 2018^23^	CT	456	EAT volume Mean HU	MACE	HR: 4.6 (95% CI: 1.6-13.1) HR: 0.8 (95% CI: 0.7-0.9)
Picard et al, 2014^19^	CT	970	EAT max thickness on LVLW>2.8 mm	CAD	OR: 1.46 (95% CI: 1.03-2.08)
Brandt et al, 2022^31^	CCTA	117	EAT volume	MACE	HR: 2.41 (95% CI: 1.08-4.72) c-index = 0.72
Uygur et al, 2021^32^	CCTA	127	EAT volume EAT volume >123.2 ${\text{c}\text{m}}^{3}$	MACE for patients with type 2 diabetes	OR: 1.027 (95% CI: 1.01-1.04) AUC = 0.82
Demircelik et al, 2014^20^	CCTA	131	EAT max thickness on RVAW	Obstructive CAD	AUC = 0.715

Multiple studies have demonstrated that EAT volume, mean HU, and thickness are predictive of MACE or CAD. Most entries should be self-explanatory. Previous studies have not included a detailed analysis of engineered features, as we have done in this paper.

AUC = area under the receiver-operating characteristic curve; LVLW = left ventricle lateral wall; RVAW = right ventricle anterior wall.

In this preliminary investigation, we sought to use AI to predict MACE using features derived from EAT in noncontrast CTCS images. This includes automated deep-learning based EAT segmentation, extraction of novel features, Cox time-to-event modeling, analysis of high-risk features, and a combined EAT fat-omics model for MACE prediction.

## Methods

### Data acquisition

A total of 400 patients without known coronary disease (including myocardial infarction, stable or unstable angina, significant coronary stenosis, or coronary revascularization) were identified from a clinical cohort undergoing clinical CTCS for risk assessment between 2014 and 2020. All CTCS scans were ECG-gated, noncontrast acquisitions, with standardized scanner settings of 120kVp. In CTCS imaging, exposure is adjusted to account for body habitus to maintain image quality. The mA is either adjusted automatically or by a technician. The mA values from 110 to 661 mA with an exposure time of 280 ms, giving mAs values ranging from 25 to 185 mAs for a Brilliance iCT Philips scanner with a rotation time of 0.33 seconds. There were some other scanners with similar characteristics. A filter back projection was used for the reconstruction. The slice thickness was 2.5 mm, and the nominal in-plane pixel spacing was 0.5×0.5 mm^2^. A standard CTCS scan contains ∼50 slices of 512 × 512 voxels.

### EAT segmentation

We emphasized accurate segmentation of cardiac fat depots. EAT is located inside the heart pericardium. Accurate manual segmentation is quite intensive, taking up to 2 hours per case with inter-reader and intrareader variability. We leveraged our previously developed DeepFat,^23^ a deep learning-based automatic segmentation method, which expedited the process and improved consistency. For the EAT segmentation, we reduced image noise by applying a 2-dimensional median filter with a kernel size of 3 × 3 voxels. Our DeepFat algorithm used various preprocessing steps (window leveling and RGB channel usage), achieving superior accuracy and robust interoperator repeatability. In our previous publication,^24^ we analyzed accuracy as compared to manual segmentation (Dice = 88.52% ± 3.3%) and interoperator repeatability (analyst1 vs analyst2: R = 0.988, *P* < 0.001), while comparable performance was observed when DeepFat was evaluated against both analyst1 (R = 0.985, *P* < 0.001) and analyst2 (R = 0.973, *P* < 0.001), indicating that our DeepFat algorithm performed as good as expert analysts. In this study, we ran DeepFat to get an initial segmentation and manually corrected any errors. This semiautomated process greatly reduced the operator time from 2 hours per image volume for fully manual analysis to a much more reasonable time (10-20 min). The detailed pipeline of automatic segmentation is illustrated in Central Illustration.

**Central Illustration:**
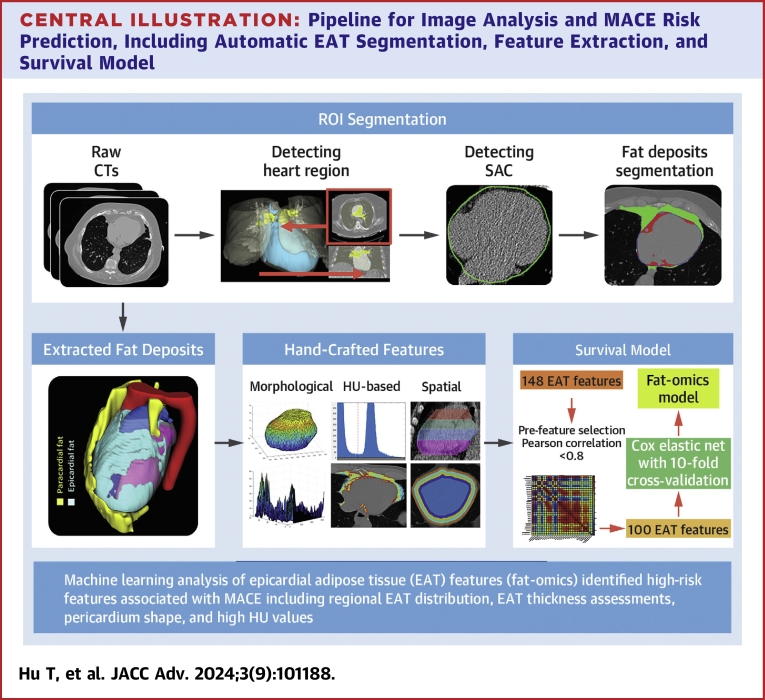
**Pipeline for Image Analysis and MACE Risk Prediction, Including Automatic EAT Segmentation, Feature Extraction, and SurvivalModel**

### Feature engineering

**Figure 1:**
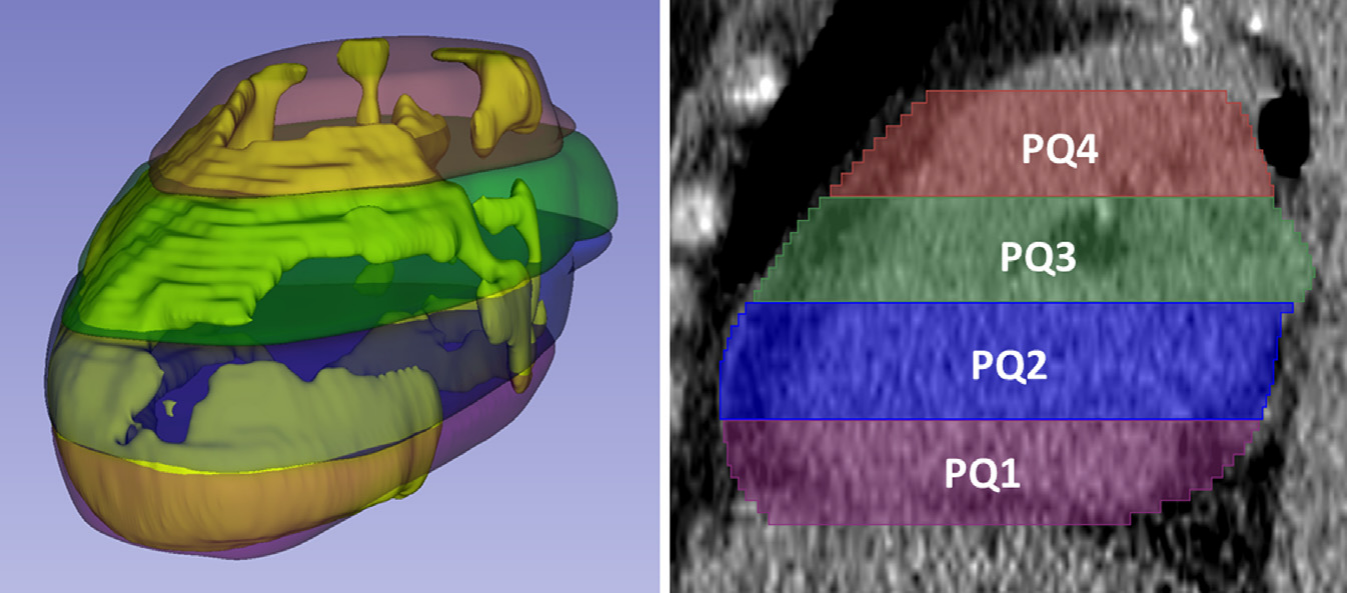
**Slab Volumes for Localizing the Spatial Distribution of Fat** The heart was divided into 4 equally thick Slabs, each composed of consecutive axial image slices. Slabs extending from the lowermost to the uppermost parts of the heart, are labeled PQ1 to PQ4. This slab-based approach allowed assessment of EAT features in different parts of the heart.

We created pathophysiological-inspired, hand-crafted features, giving us 148 features from EAT. Features were organized into 3 categories: morphologic, HU, and spatial features (Central Illustration). Examples of morphologic features are volume, principal axis lengths, and EAT thickness. HU-based features included statistical measurements, such as HU min, max, mean, skewness, and histogram bins. To analyze the spatial distribution of EAT within the heart region, we subdivided the heart region into 4 equally thick (axial) slabs of image slices from top to bottom (Figure 1) and 4 equidistant ribbons from outside to inside (Central Illustration).

**Figure 2:**
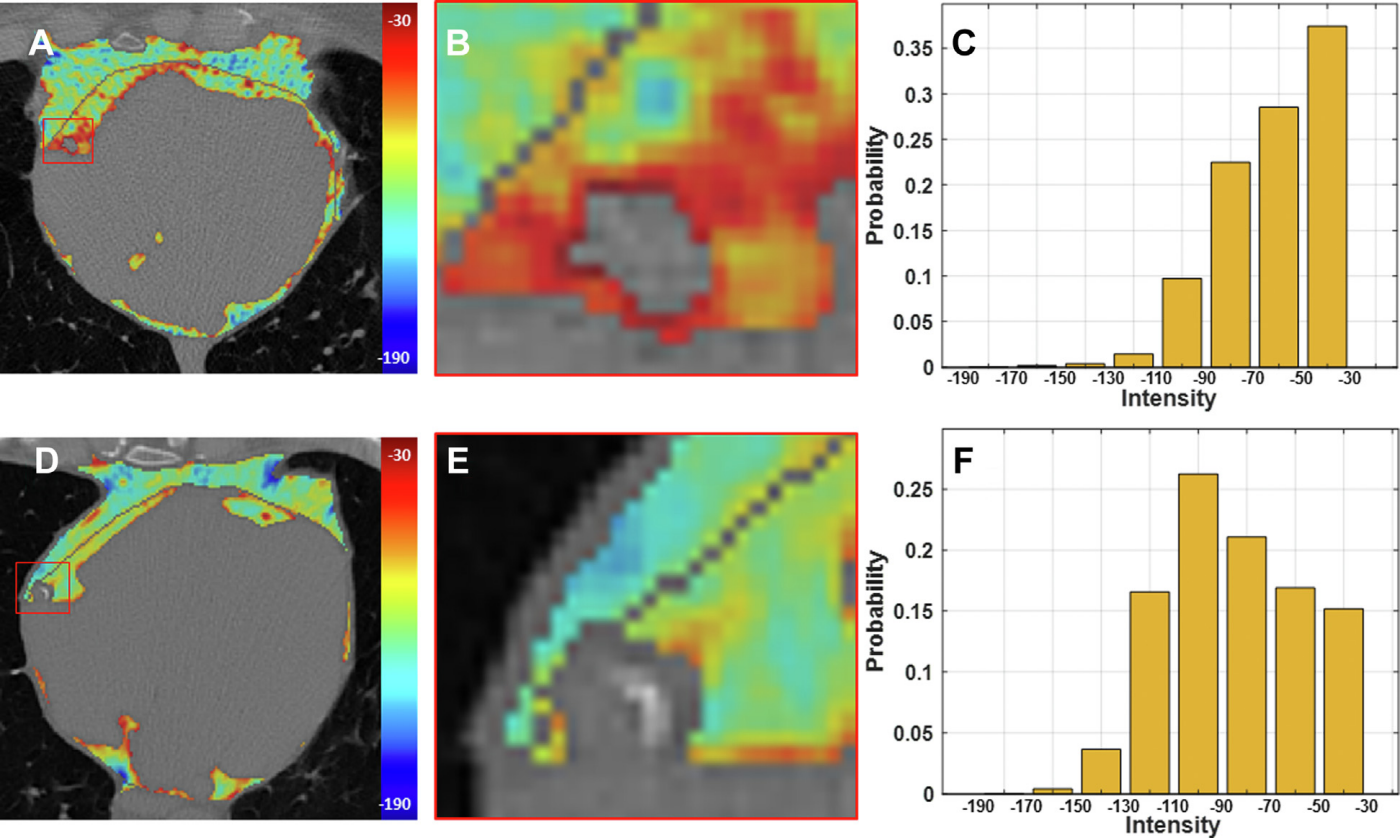
**Example EAT CT Intensities (HU Values) in Patients With and Without MACE** (A and D) EAT HU (inside the pericardium in red) is elevated as compared to PAT HU (outside the pericardium). Zoomed views show that the specialized EAT around the RCA (called PCAT) has elevated HU as compared to PCAT outside the black pericardium boundary. Importantly PCAT is clearly elevated in E relative to B. (C and F) Present normalized HU distributions for EAT. For the MACE patient (C), There is a conspicuous shift towards higher HU values with 37% of values within the [-50, -30 HU] range, as compared to 15% for the no-MACE patient.

**Figure 3:**
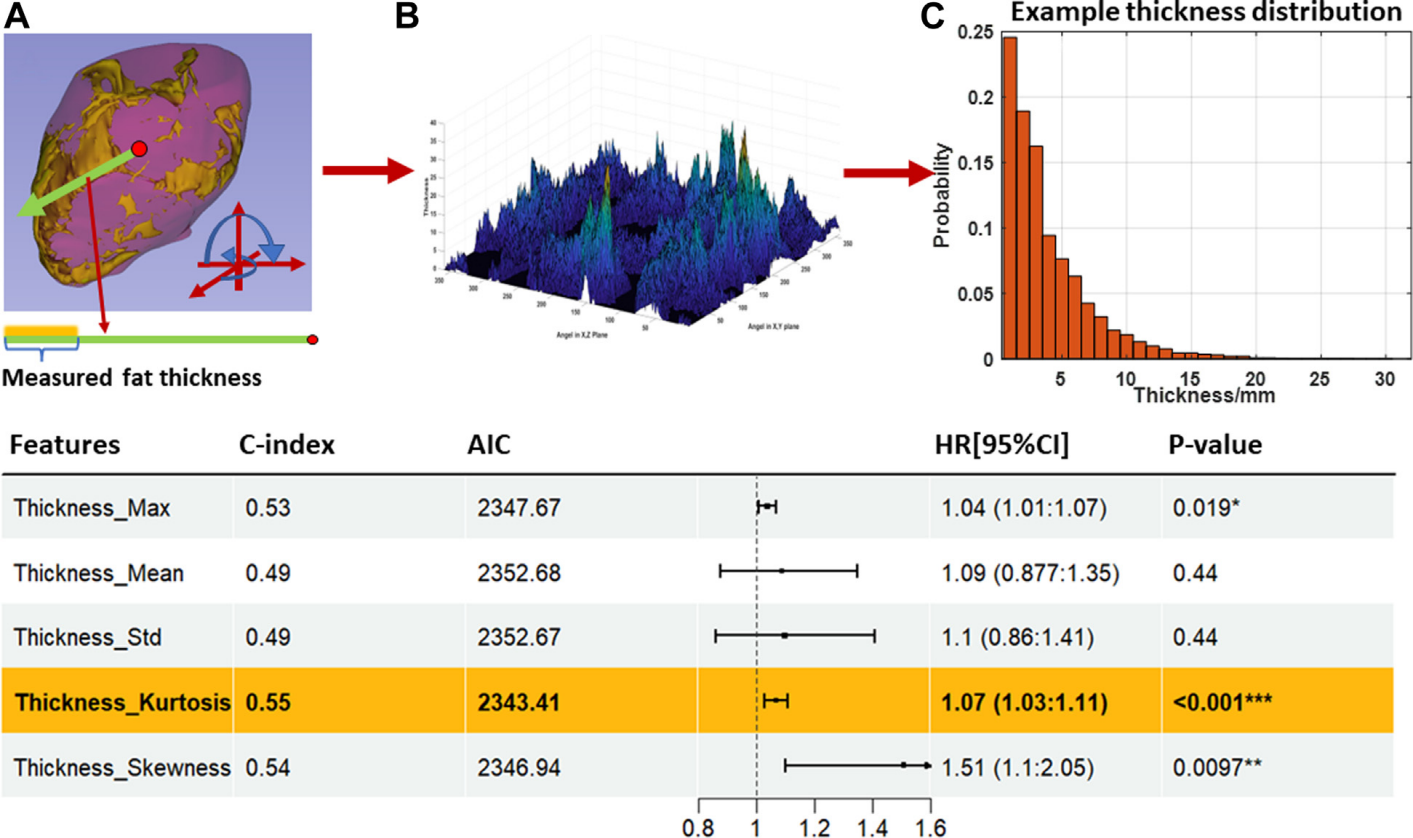
**Univariable MACE Cox Modeling Analysis of EAT Thicknesses** Euclidian “thickness” was measured along a ray emanating from the center of mass of the pericardial Sac volume (A). The ray was swept along all possible angles at 1-degree increments to create a 2D array of thickness values (B), Which were then gathered to create a histogram (C). On the bottom, we present univariable Cox model results for features derived from thickness histograms. Thickness kurtosis and skewness gave significant HRs whereas other features did not, suggesting that larger thickness values have risk associated with them. See Table 3 for other details. *P* values are star-coded based on the significance levels as follows: (<0.001 as ∗∗∗, <0.01 as ∗∗, <0.05 as ∗).

To use features from the EAT thickness distribution, we developed a novel algorithm (see later in Figure 3A) that measured EAT thicknesses across the EAT 3D surface. Along each ray from the centroid of the heart to the pericardium surface, the algorithm measured the Euclidean distance between the first and last intersected fat voxels. Rays scan the whole 3-dimensional sac surface with a 1-degree shift (ie, 360-degree in the XY-plane and 180-degree in the Z-direction). This method provided 64,800 EAT thickness measurements for each patient. Subsequently, we computed statistical features from the distribution (eg, mean, max, skewness). Additionally, we divided the thickness measurements into 4 fixed histogram bins (each 8-mm wide), as determined by the spread of thickness observed across all persons. This allowed us to identify specific thickness ranges that may be particularly relevant for the prediction of MACE. This method provides a much more detailed and comprehensive assessment of EAT thickness as compared to previous studies.

We created features to analyze EAT elevated HU values, thought to be an indicator of adipose inflammation.^10^ This provided a more detailed analysis than the standard approach of computing mean HU,^21^^,^^22^ We used statistical measurements of the distribution of HU values, including extremes (max, min), peakedness (via kurtosis), and asymmetry (via skewness) of the HU distributions. In addition, we analyzed 4 and 8 histogram bins of HU values and extracted statistical metrics for each defined range. Bin sizes were fixed across the cohort of patients.

In addition, we also created features associated with the spatial distribution of EAT. For each patient, we divided the number of slices into 4 equal quartiles, dividing the EAT volume into 4 slabs from bottom to top (Figure 1). Similarly, for each patient, we used the major principal axis length to divide EAT volume into 4 equidistant “shells” from outer to inner (Central Illustration). This division was performed using morphological operations. Altogether, these processes divided the EAT volume into 4 subregions axially and 4 shell radially. For each subregion, we computed features (eg, morphologic and HU-based). Example subregion features are: EAT volume for the topmost axial subregion of the heart and HU analyses for the outermost shell. Compared to the state-of-the-art methods,^16^, ^17^, ^18^, ^19^, ^20^, ^21^, ^22^ our proposed novel feature extraction methods create a much more comprehensive evaluation of EAT.

### Feature selection and evaluation metrics

We performed feature reduction to limit correlated features and overtraining. Starting with 148 EAT features, we applied maximum relevance minimum redundancy, to obtain 50 fat-omics features. Next, we used the Cox proportional hazard model with elastic regularization to select the most effective features.^25^ Specifically, we used elastic net regularization with a mixing parameter (α) of 0.8, determined using grid search, to balance the strengths of the L1 (Lasso) and L2 (Ridge) penalties to achieve optimal performance. On the training set, we used Cox proportional hazard with elastic net (cv.glmnet code). This code evaluates folds to limit effects of data selection (10-folds were used), gives guidance for hyperparameters, does elastic net feature reduction, and outputs a set of features with corresponding coefficients forming the Cox model. We settled on 15 fat-omics features. All feature and hyperparameter selections were performed on the training set. The model with fixed coefficients was then evaluated on both the training and held-out tests set using various performance metrics (eg, C-index, Akaike’s information criterion, HRs, and area under the receiver-operating characteristic curve at various time points). We also analyzed HRs for the 15-feature fat-omics model in comparison with simpler models that utilized traditional features such as EAT volume and EAT mean HU. To assess the variability in model performance due to data selection within the training set, we employed a bootstrapping technique. We fixed the 15 features, optimized a Cox model using 10-fold cross-validation with 1,000 bootstrap iterations with replacement, and determined the mean and CIs of performance metrics.

### Bottom of form

In our Cox modeling implementation, we use R software version 4.2 with package libraries glmnet for cox modeling and elastic net, riskRegression for evaluation, and mRMRe for feature reduction prior to Cox modeling. We used MATLAB, version, 2022b with Deep Learning Toolbox for DeepFat segmentation.

## Results

### Data set

Patient records were queried for incidence of MACE (4-points MACE defined as myocardial infarction, stroke, coronary revascularization, and all-cause mortality) obtained from electronic health record review. The maximum recorded follow-up observation time was 6 years with a median of 1.4 years (95% CI: −1.2 to 1.6 years). Times were measured from the time of imaging to MACE or censoring. The data set in this preliminary study was MACE-enriched (56% MACE) to improve the precision of image-derived effects. By increasing the event rate with this cohort, we enhanced the statistical power of our analysis. This aided feature creation and optimization. In addition, the increased event rate would improve Cox elastic net optimization and feature reduction reliability.

Detailed data exclusion criteria are shown in Supplemental Figure 1. In this preliminary study, we randomly selected participants with nearly equal numbers from 4 different groups depending on Agatston score and MACE occurrence (AG = 0 MACE = 0; AG = 0 MACE = 1; AG > 0 MACE = 0; and AG > 0 MACE = 1). We then excluded patients with known CAD and follow-up time of <10 days, resulting in our final cohort (N = 400). The data set was randomly divided into training/held-out testing subsets (80%/20%), respectively, while maintaining equal MACE ratio in both subsets. Clinical data such as age, sex, body mass index, and Agatston score, were also collected. Some clinical data elements were missing (eg, out of 400 patients, 339 had reported body mass index). Population characteristics are described in Table 2 and Supplemental Table 1. Please note that there is no significant difference between train/test cohorts, indicating a fair train/test split.

**Table 2:** Characteristics of the 400-Patient Cohort With an Enriched MACE Rate (56%) and With Proven EAT Segmentation

	Full Cohort (N = 400)	MACE Subcohort (n = 224, 56%)	No MACE Subcohort (n = 176, 44%)	*P* Value
Female	214 (54%)	112 (50%)	102 (58%)	-
Age, y, mean (min, max)	61 (18, 87)	63.2 (18, 87)	58.3 (21, 84)	<0.001∗∗∗
MACE events	224 (56%)	-	-	-
Observation time	641.3 (10, 2,176) day 1.8 (0, 5.9) year	505.3 (10, 2,170) day 1.4 (0, 5.9) year	814.5 (14, 2,176) day 2.2 (0, 5.9) year	<0.001∗∗∗
Average score	602.3 (0.5, 879)	952.7 (0.5, 897)	156.3 (0.3, 611)	<0.001∗∗∗
Zero average score	204 (51%)	116 (52%)	88 (50%)	-
EAT volume (cm^3^)	120.7 (20.5, 399.4)	127.4 (26.8, 399.4)	112.2 (20.5, 300.1)	0.004∗∗
SAC volume (cm^3^)	774.9 (391, 1,452.1)	791.3 (464.2, 1,312.1)	754 (391, 1,452.1)	0.004∗∗
EAT max thickness (mm)	19.7 (5.5, 31.3)	20.1 (7.5, 31.3)	19.2 (5.5, 28.8)	0.003∗∗

Values are n (%) or mean (min, max).

*P* values are star-coded based on the significance levels as follows: (<0.001 as ∗∗∗, <0.01 as ∗∗, <0.05 as ∗).

Several assessments are significantly different between the groups.

EAT = epicardial adipose tissue; MACE = major adverse cardiovascular event.

### Feature analysis

We performed a comprehensive feature analysis to identify the most influential features for predicting MACE within each feature category. We started with the spatial distribution of EAT. In Table 3, we compared the predictive ability of EAT volume and each slab subregion using univariable Cox models. Total EAT volume has a positive, significant HR but a relatively low C-index (C-index = 0.53, *P* = 0.005). The volume in the top slab of the heart has a higher risk as compared to other slabs. It has a positive HR with a CI above 1.0 and is the most predictive (C-index = 0.56, *P* = 0.001) with the lowest Akaike’s information criterion. The top slab is consistent with the location of the preponderance of pericoronary adipose tissue. We performed similar volume analyses on shell subregions (Supplemental Table 2). The volume in the outermost subregion shows the most significance with a C-index of 0.56 (*P* < 0.001).

**Table 3:** Univariable Cox Modeling of EAT Volumes, Including Contributions From Different Slabs

Features	C-Index	AIC		HR (95% CI)	*P* Value
EAT_vol	0.53	2345.81		1.20 (1.06-1.36)	0.0046∗∗
Vol_PQ1	0.53	2349.07		1.15 (1.01-1.30)	0.037∗
Vol_PQ2	0.53	2345.77		1.20 (1.06-1.35)	0.0037∗∗
Vol_PQ3	0.52	2349.05		1.15 (1.01-1.30)	0.033∗
Vol_PQ4	**0.56**	**2343.41**		**1.23 (1.09-1.39)**	**0.0012∗∗**
					

Each row includes the C-index, AIC, HR with a CI, and *P* value for a feature. Total EAT_vol is significant, but despite its prominence in the literature, its C-index is only 0.53. EAT in the top of the heart (ie, Slab PQ4), is the most significant and gives a C-index of 0.56. Notably, this slab coincides with the region subjected to pericoronary adipose tissue analysis. For this feature analysis, all data were used with coxph (survival R package), which gave CIs and *P* values. *P* values are star-coded based on the significance levels as follows: (<0.001 as ∗∗∗, <0.01 as ∗∗, <0.05 as ∗).

EAT = epicardial adipose tissue; MACE = major adverse cardiovascular event.

We also analyzed the role of HU values on MACE prediction. In Figures 2A and 2D, we showed spatial distributions of HU values for example patients with and without a MACE, respectively. In the PCAT surrounding the RCA (Figures 2B and 2E), the patient with MACE had clearly elevated HU values as compared to the patient without MACE, consistent with reports in the literature suggesting that elevated HU in PCAT is indicative of inflammation and increased risk of MACE.^10^^,^^11^ In the histograms (Figures 2C and 2F), the patient with MACE had a decided shift to high HU values relative to the patient without MACE, again emphasizing the effect of HU values. Using similar univariable Cox analyses, we further analyzed HU features across individuals (Table 4). The mean HU of EAT has an overlapping HR across 1, indicating it is a poor predictor of MACE. HR increases and becomes more significant with higher HU ranges. The volume of voxels within −50 to −30 HU, the highest bin of HU, is the most significant feature, emphasizing the importance of elevated HU values. Negative HU skewness is also significant, again emphasizing the importance of high HU values. In addition to using the volumes of tissue within each range, we normalized histograms and obtained a probability of being within a range of HU values. Results (C-index = 0.55, HR: 1.13, 95% CI: 1.0-1.29; *P* = 0.05) were slightly worse compared to those obtained with absolute volumes (Supplemental Table 3). Both normalized and unnormalized features were included in our starting comprehensive feature set.

**Table 4:** Univariable MACE Cox Modeling Analysis of EAT HU Values, Including Analysis of Different HU Ranges

Features	C-Index	AIC		HR (95% CI)	*P* Value
EAT_vol	0.53	2345.81		1.20 (1.06-1.36)	0.0046∗∗
EAT_HUmean	0.48	2353.26		1.00 (0.98-1.02)	0.97
vol_190_170	0.55	2352.59		1.05 (0.942-1.16)	0.40
vol_170_150	0.54	2351.59		1.07 (0.969-1.19)	0.18
vol_150_130	0.52	2351.07		1.09 (0.976-1.23)	0.12
vol_130_110	0.51	2349.98		1.12 (0.997-1.26)	0.056
vol_110_90	0.51	2349.10		1.15 (1.01-1.30)	0.034∗
vol_90_70	0.53	2347.57		1.18 (1.03-1.35)	0.015∗∗
vol_70_50	0.56	2343.63		1.23 (1.08-1.40)	0.0015∗∗
vol_50_30	0.57	2338.47		1.29 (1.14-1.47)	<0.001∗∗∗
Negative_skewness	0.56	2350.00		1.13 (1.00-1.27)	0.049∗
					

In rows 3-10, the volume of voxels within an HU range is analyzed (eg, Vol_190_170 corresponds to the volume of EAT having HU values between −190 and −170 HU). HR values increase and become more significant with higher HU ranges, with Vol_50_30, being the most significant. The histogram metric, negative_skewness allows one to capture the tendency to high HU values and is also significant. The mean HU value, EAT_HUmean, has HR overlapping 1.0 with a poor *P* value, indicating that it is not a good predictor of MACE. See Table 3 for other details. *P* values are star-coded based on the significance levels as follows: (<0.001 as ∗∗∗, <0.01 as ∗∗, <0.05 as ∗).

We analyzed the role of EAT thicknesses on MACE prediction using univariable Cox models (Figure 3). Figure 3A shows the process of measuring EAT thickness, where all thicknesses were determined along rays originating from the centroid of the pericardium to the 3-dimensional surface. We collected all measurements (Figure 3B) and analyzed them in terms of histograms (Figure 3C). In the table, the mean thickness is not a good predictor (C-index = 0.49, HR: 1.09, 95% CI: 0.87-1.35; *P* = 0.44]). Maximum thickness, histogram kurtosis, and histogram skewness give significant HRs. Notably, the largest thickness bin (24-32 mm) is most significant (C-index = 0.53, HR: 1.19, 95% CI: 1.05-1.35; *P =* 0.006) among all bins (Supplemental Table 4). Together, investigations indicate that large thickness values were important for MACE prediction.

**Figure 4:**
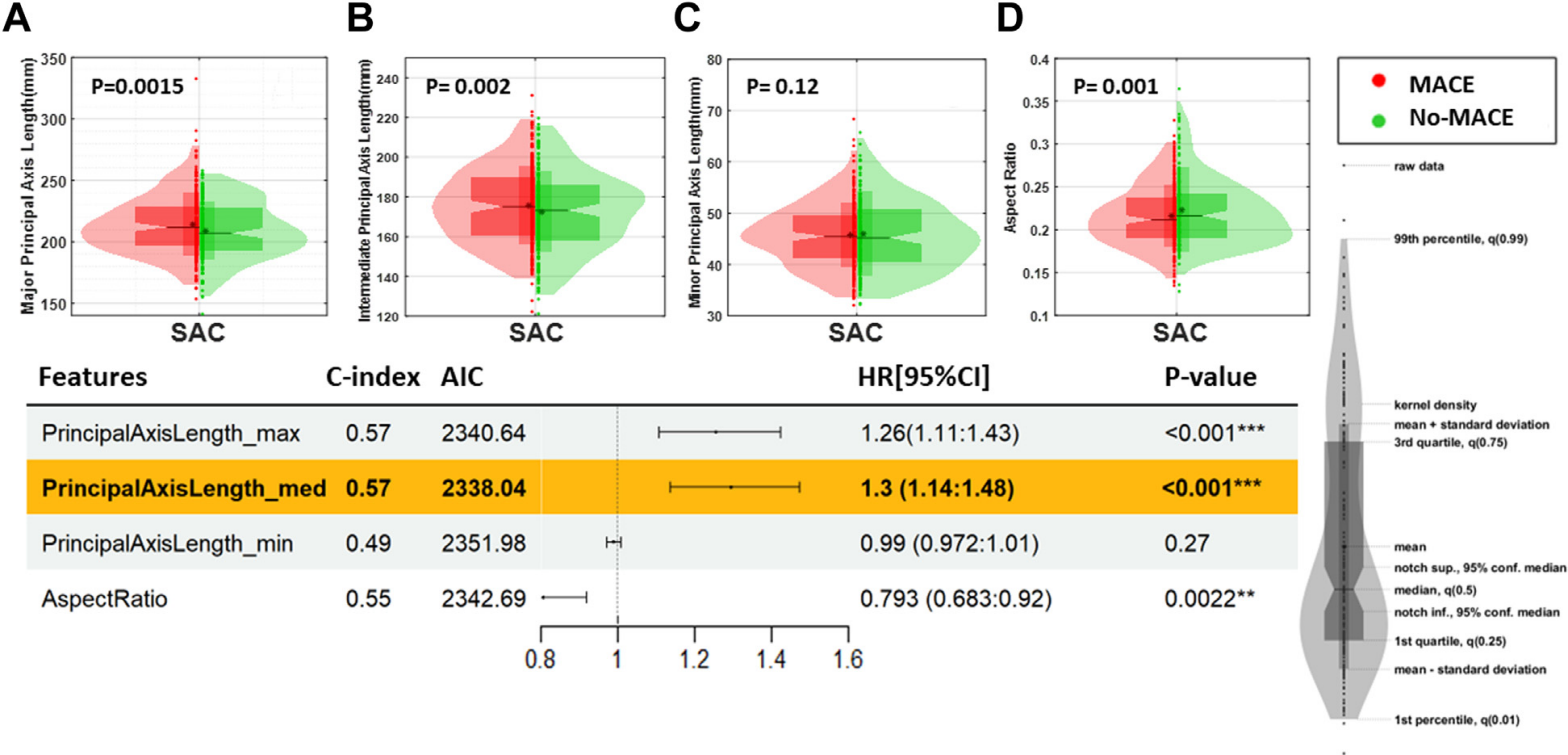
**Univariable Shape Features Analysis as Assessed by Principal Axes of the Pericardial Sac Volume** Along the top (A to C), we show violin plots of the major, intermediate, and minor axes for MACE (red) and no-MACE (blue) groups, where p values were obtained from a 2-sample t-test. Aspect ratio is shown in D. The major (A) and intermediate (B) axes lengths are significantly different for MACE and no-MACE. These findings are corroborated by the univariable Cox analysis in the table at the bottom. The major and intermediate axes lengths give significant HR all above 1 whereas the minor is not. *P* values are star-coded based on the significance levels as follows: (<0.001 as ∗∗∗, <0.01 as ∗∗, <0.05 as ∗).

We analyzed the role of the shape of the heart as assessed from the pericardium segmentation on MACE prediction (Figure 4). Figures 4A to 4D show the distribution of heart’s principal axes and aspect ratio across MACE and no MACE groups. We found that the lengths of the major and intermediate axes were the most significant, with the minor axis length having little effect. This suggests that a large, flat sac volume is a risk factor for MACE.

### HRs and model prediction performance

In Table 5, we compared Cox models designed from combinations of important subsets of fat-omics features to our proposed 15-feature fat-omics model. We include results on the training, bootstrap validation, and held-out testing cohorts. For baseline comparisons, we included predictions based upon (ie, EAT_vol, mean_HU, and thickness_Max). These gave test C-index values not much above guessing (0.5). Hazard ratios for 1.2 and 1.1 were close to 1.0 but significant. EAT means HU was not significant. When we combined these 3 “traditional” features in line 4, prediction was improved giving a test C-index of 0.6, suggesting independence of the features. Prediction from only 3 high-risk features previously discussed (Vol_PQ4, Pro_50_30, and Thickness_Kurtosis) further improved performance (test C-index = 0.64), and all these features gave high HR values and were significant. Finally, our full fat-omics model with the 15 fat-omics features identified in Supplemental Tables 5 and 6 gave by far the best results on this data set. It gave the best performance values in each column, including test C-index and 2-year area under the receiver-operating characteristic curves of 0.69 and 0.70, respectively. Please note that both training and testing C-index for all models are within the range of the bootstrap validation spread, suggesting that results are not overly dependent on the data selected.

**Table 5:** Risk Prediction From Our Aggregated Fat-Omics Model as Compared to Predictions From Subsets of Features, Including “Traditional” Ones

Model Specification		Training (N = 320)					Testing (N = 80)	
Model	Feature	HR (95% CI); *P* Value	C-Index	AUC 2-y	AIC	Bootstrap Validation	C-Index	AUC 2-y
						C-Index [95% CI]		
EAT vol	Ln (EAT_vol)	1.2 (1.0-1.4); 0.026∗	0.53	0.53	1895.40	0.54 (0.48-0.56)	0.53	0.50
EAT mean HU	EAT_mean_HU	1 (0.98-1.02); 0.99	0.49	0.48	1901.07	0.53 (0.48-0.56)	0.55	0.57
EAT thickness	Thickness_Max	1.1 (1-1.1); 0.007∗∗	0.54	0.56	1893.84	0.53 (0.48-0.57)	0.51	0.52
EAT traditional features	Ln (EAT_vol)+ EAT_mean_HU+ Thickness_Max	1.6 (0.86-2.9); 0.142 1.0 (1-1.1); 0.021∗ 1.1 (1-1.1); 0.069	0.57	0.60	1891.21	0.58 (0.52-0.62)	0.60	0.58
EAT high-risk features	ln(Vol_PQ4) + Pro_50_30 + Thickness_Kurtosis	2 (1.3-1.7); <0.001∗∗∗ 28 (1.3-588); 0.032∗ 1.04 (1.01-1.09); 0.044∗	0.6	0.66	1883.44	0.60 (0.55-0.65)	0.64	0.69
Fat-omics	**Fat-omics (15 features)**^a^	2.7 (2.1-3.5); <0.001∗∗∗	**0.66**	**0.72**	**1864.66**	**0.65 (0.59-0.71)**	**0.69**	**0.70**

*P* values are star-coded based on the significance levels as follows: (<0.001 as ∗∗∗, <0.01 as ∗∗, <0.05 as ∗).

In lines 1 to 3, we evaluated EAT volume, mean HU, and maximum thickness. Despite their appearance in previous publications, the predictive capability of each of these features was marginal. The combination of these features improved performance (line 4). A Model with our 3 previously identified high-risk features improved prediction (line 5). Each of these high-risk features had a positive effect HR and significant *P* values, highlighting their importance. The fat-omics model surpassed all other assessed models with the highest C-index, 2-year AUC, and lowest AIC for both the training and held-out testing sets. To evaluate the spread of results due to sampling, bootstrap validations on the training set (1,000 iterations) are reported. The fact that both the training and the held-out test results are within the range of bootstrap validation indicates that results did not overly depend on selected data.

a The reported HR represents the effect of the combined fat-omics score. This score is calculated as the summation of each selected feature multiplied by its respective coefficient. The single HR thus reflects the overall impact of the aggregated fat-omics features on the prediction of MACE. For details HRs about the selected features, please refer to Supplemental Table 4.

**Figure 5:**
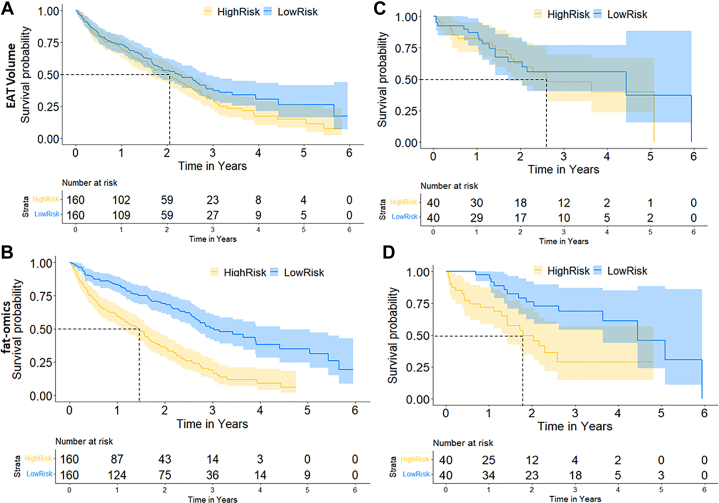
**MACE Risk Stratification (Kaplan-Meier Plots) for Our MACE-Enriched Data Set** We analyzed both the EAT_vol (A, C) and fat-omics (B, D) models for the full cohort, including training data (left column) and the much smaller testing cohort (right column). We stratified high- and low-risk cases on the median risk and then computed Kaplan-Meier. Mean test survival times for the high-risk groups are 1.78 years and 2.62 years for the fat-omics and EAT_vol, respectively. The fat-omics outperformed EAT_vol in both training and testing sets in terms of NRI. Please note that we use a small MACE-enriched cohort to engineer features. These small probabilities of MACE-free survival will not hold for the general population.

In addition, we compared the ability of the combined 15-feature fat-omics model to stratify high- and low-risk groups to that of the traditional EAT_vol Cox model (Figure 5). In the testing cohort, the 15-feature fat-omics model resulting in a significant reclassification of risk beyond EAT volume at year 2 (training: $NRI_{Categorical}=0.259$ [95%CI:0.10-0.37;P<0.001] ($NRI_{Nonevent}=0.153$, and $NRI_{Event}=0.106$), and testing: $NRI_{Categorical}=0.101\;\left[95\%\;CI:\;-0.17\;\text{t}\text{o}\;0.37;\;P=0.463\right]$ $(NRI_{Nonevent}=0.044\;\text{and}\;NRI_{Event}=0.057$). The nonsignificant *P* value in the testing cohort can be attributed to the small size of this cohort, which comprised only 80 patients.

**Figure 6:**
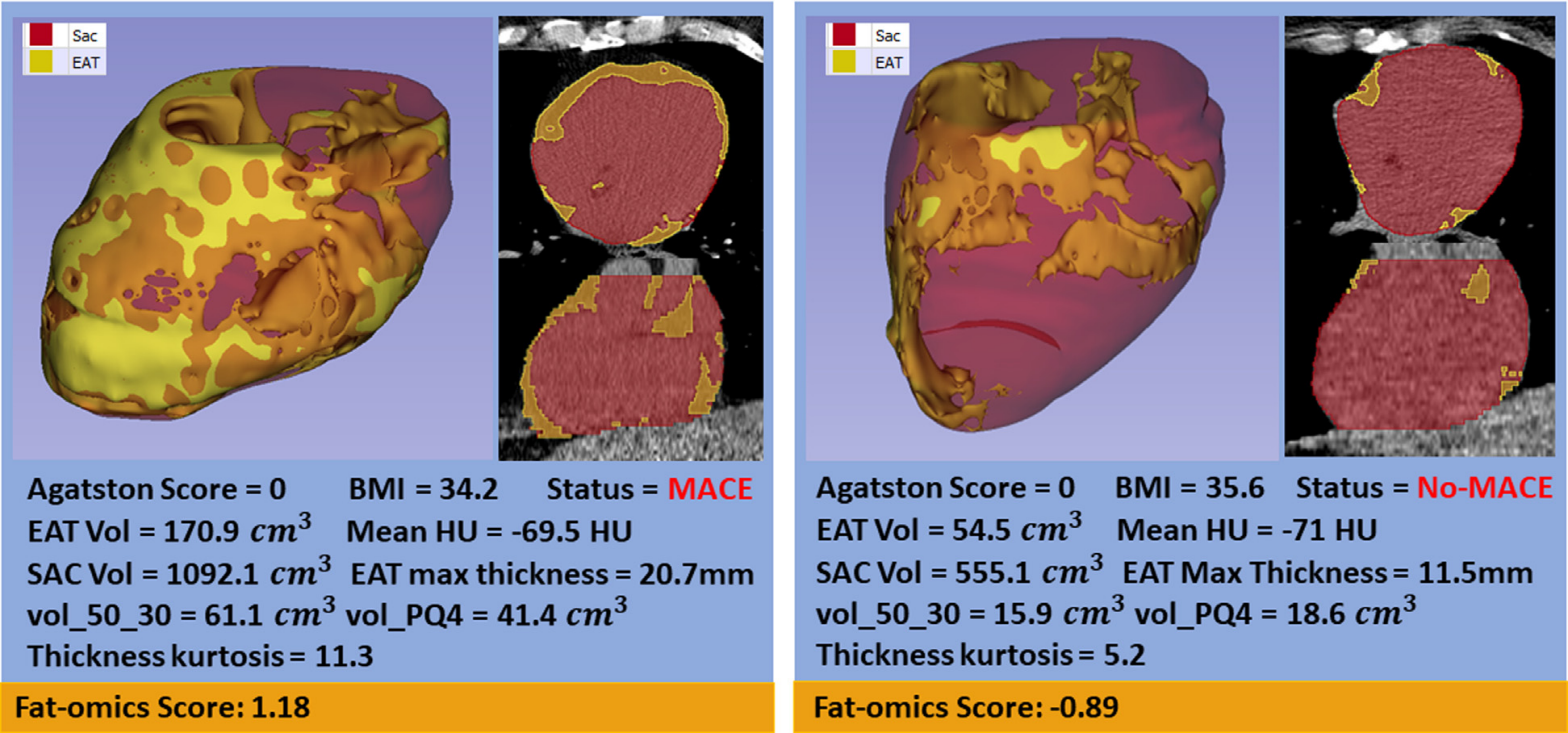
**Comparison of Cases Having Similar BMIs and Agatston of Zero but Having Much Different EAT Profiles** On the left, the heart has higher EAT, Sac, mean HU, and maximum EAT thickness than for the “Lean” heart on the right. Our identified high-risk features (vol_50_30, vol_PQ4, and Thickness Kurtosis) shows even more difference. Applying the fat-omics model, the risk for the left heart was 3-times that for the one on the right. In the case of the left heart, there was a MACE later in the study.

To highlight the importance of fat-omics results, we showed 2 exampled cases (Figure 6). Both cases have similar body mass index and Agatston calcium scores of zero. The heart on the left has much higher EAT volume and vol_50_30 values than the one on the right. The 15-feature fat-omics model predicted 3 times the risk for the heart on the left than the one on the right. There was a MACE for the heart on the left but not the right.

## Discussion

To the best of our knowledge, we have created the first-ever machine learning comprehensive analysis of EAT features (our fat-omics) for MACE prediction. Previous reports identify that EAT volume^20^, ^21^, ^22^ and, EAT HU^21^^,^^22^ are associated with MACE, and that the thickness of the right ventricular anterior free wall is correlated to obstructive CAD.^19^ We used features like these and investigated our hand-crafted features in analyses. Our comprehensive fat-omics risk prediction model with 15 hand-crafted features was much improved as compared to other models evaluated. For example, the 15-feature fat-omics model gave testing C-index/2-year area under the receiver-operating characteristic curve values of 0.69/0.70, while EAT volume and mean HU yielded only 0.53/0.50 and 0.49/0.55, respectively. High-risk features in fat-omics included assessments of regional EAT distribution, EAT thickness assessments, pericardium shape, and high HU values, suggesting that EAT is indeed a risk factor for MACE. It appears that much more can be gleaned from a more detailed assessment of EAT than has been done in the past. This will lead to a path for important studies combining fat-omics features with coronary calcification assessments. Potentially, this will lead to much-improved risk prediction as compared to the Agatston score, the only current standard clinical assessment from CTCS images.

We identified multiple novel features important for MACE prediction. These include the EAT volume in the top quartile slab of the heart, the EAT volume of voxels within −50 to −30 HU, thickness kurtosis, and the major principal axis length. Importantly, the volume of voxels between −50 and −30 HU (vol_50_30) was the most MACE informative feature. Fat inflammation is linked to high HU, presumably due to morphologic changes in adipocytes,^24^ inhibition of local adipogenesis,^10^ and increase in vascular permeability.^24^ See Figure 2 for an example figure showing increased HU values with MACE. The increase in the highest EAT HU values is elegantly captured with vol_50_30. The attribute of high HU values is also captured by negative skewness of the HU distribution, again found to be a significant risk factor of MACE in time-to-event modeling.

There are some limitations to our preliminary study. Most importantly, we used a small MACE-enriched cohort. This was done so that we could emphasize feature engineering on a data set with expert proven, accurate EAT segmentations. By using a MACE-enriched cohort, we emphasize the importance of imaging features with a reduction of the uncertainty obtained with low event rate time-to-event modeling. Another limitation is that the MACE-free survival curves (Figure 5) are dedicated for our MACE-enriched data set only and not for a general population. Results reported here are for 4-point MACE, which includes revascularization. When we changed the outcome to 3-point MACE, without revascularization (Supplemental Table 7), we obtained a similar performance (testing C-index = 0.66). In addition to revascularization, as others have done, our definition of 4-point MACE includes “all-cause mortality,” which might introduce potential bias since not all deaths are attributable to cardiovascular disease. Furthermore, although we have identified explainable, very promising, novel features, there are always more features that could be created. Deep-learning approaches may be able to capture more complex features and improve the predictive power of the model. However, the explainability of our results (eg, the contribution of high HU adipose volume) encourages the hand-crafted feature approach. Note that our fat-omics features were created by us and are not found in existing radiomics-style libraries.

In conclusion, our study highlights the potential of applying a detailed image-based analysis of EAT for improving the prediction of cardiovascular risk. We have demonstrated that this can be done opportunistically in low-cost (no-cost) CTCS examinations. It will be most interesting to combine AI analysis of adipose depots and coronary calcifications for risk prediction. Given preliminary reports of the independence of these assessments,^26^ such an analysis holds promise. It will be interesting to determine if risk prediction from EAT is useful for identifying those patients with zero calcium who go on to have a MACE. There are drugs like GLP1 agonists and SGLT2 inhibitors,^27^ which tend to reduce epicardial adipose tissue,^28^ suggesting a mechanism for cardioprotection that could be further analyzed with a fat-omics model. Future studies are needed to validate our findings and to explore the use of more complex machine-learning approaches to improve the predictive power of the model.

PERSPECTIVES **COMPETENCY IN MEDICAL KNOWLEDGE:** Recent studies only used basic EAT assessments to predict risk of MACE. Detailed assessments of EAT in CTCS images can enhance the prediction of MACE by capturing pathophysiology of EAT. These preliminary findings suggest the potential of refined and explainable EAT evaluations to improve cardiovascular risk prediction.

**TRANSLATIONAL OUTLOOK:** A broader and more general population is needed to validate our findings and to assess the added value on top of calcium scoring.

## Funding support and author disclosures

Human subject research has been done under an IRB of Case Western Reserve University (CWRU) and University Hospitals Health Systems (UHHS), Cleveland, OH. CT calcium score images were acquired at UHHS, de-identified, and shared under a data use agreement. Research was supported by the 10.13039/100000050National Heart, Lung, and Blood Institute through grants R01HL167199, R01HL165218, and R44HL156811. The content of this report is solely the responsibility of the authors and does not necessarily represent the official views of NIH. The only potential conflicts of interest relevant to the technology described herein are pending CWRU and UH patents to analyze CT calcium score images. This information has been disclosed to CWRU, and PI DLW has an approved CWRU plan for managing any potential conflicts. The authors have reported that they have no relationships relevant to the contents of this paper to disclose.
