# Supplementary material for: Artificial Intelligence Prediction of Cardiovascular Events Using Opportunistic Epicardial Adipose Tissue Assessments From Computed Tomography Calcium Score
Source: JACC Adv. 2024 Aug 28;3(9):101188. doi: 10.1016/j.jacadv.2024.101188 (PMC11450955; doi:10.1016/j.jacadv.2024.101188)

**SUPPLEMENTAL APPENDIX**

**1 Detailed feature engineering**

Fat-omics involves precise measurements of heart structure and epicardial adipose tissue (EAT). The heart is equally divided into four axial slabs (Positional Quartiles (PQ)) from the top (PQ1) to the bottom (PQ4). EAT Hounsfield unit quartiles (HQ), ranging from 1 to 4, categorize HU values into bins: HQ1 includes values from -190 to -150, HQ2 from -150 to -110, HQ3 from -110 to -70, and HQ4 from -70 to -30. Spherical regions (SR) consisted of equidistant radial shells from the outside (SR1) to the inside (SR4) of the heart. We divided the thickness measurements into four fixed histogram bins (each 8mm wide).

- 1. ***Structural features***
- **Total_SAC_Volume_Cm3** (total pericardial sac volume in cm^3^)
- **WHV** (whole heart volume: pericardial sac volume – EAT volume)
- **PrincipalAxisLength_max** (major principal axis length of the pericardial sac)
- **PrincipalAxisLength_min** (minor principal axis length of the pericardial sac)
- **PrincipalAxisLength_med** (intermediate principal axis length of the pericardial sac)
  1. ***EAT features***
- **Total_Volume_Cm3** (EAT volume in cm^3^)
- **Total_Normalized_EAT** (EAT volume/pericardial sac volume)
- **Total_HU<<stats>>** (<<mean, median, max, min, kurtosis, skewness>> of the HU of EAT)
- **Thickness_<<stats>>** (<<mean, median, max, min, std>> of the EAT thickness)
- **NormThickness_<<stats>>** (<<mean, median, max, min, std>> of the EAT thickness divided by the corresponding radius in the same direction)
- **Thickness_bin<<number>>_Pro (**probability of fixed thickness bins**)**
- **<<stats>>HU_PQ<<number>>** (<<mean, median, max, min, std, kurtosis, skewness>> of the HU in each positional quartile <<number>> [1-4])
- **Vol_PQ<<number>>** (volume of EAT in cm^3^ in each position quartile <<number>> [1-4])
- **<<stats>>HU_HQ<<number>>** (<<mean, median, max, min, std, kurtosis, skewness>> of EAT HU in each HU bin HQ <<number>> [1-4])
- **PixelCount_HQ<<number>>** (number of voxels of EAT in HQ <<number>> [1-4])
- **Probability_HQ<<number>>** (probability of EAT Voxels with HU in HQ <<number>> [1-4])
- **Vol_HQ<<number>>** (EAT volume in each HQ <<number>> [1-4])
- **Pro_HQ<<HU range>>** (probability of EAT voxels in each HQ <<number>> [1-4])
- **SR<<number>>_Pro_<<HU range>>** (probability of EAT voxels in HU ranges <<HU range>> ([-190, -170], [-170, -150], [-150, -130], [-130, -110], [-110, -90], [-90, -70], [-70, -50], [-50, -30]) in each spherical region <<number>>[1-4])

**2 Dataset, Radiomics feature analyses, and performance analysis**

We provided additional information about our dataset creation and patient characteristic across train/test split. We also included detailed analyses into fat-omics features that correlate with the risk of major adverse cardiovascular events (MACE). These analyses included a range of EAT features, stratified by spherical regions (SR), Hounsfield Unit (HU) bins, and fixed thickness bins, to uncover the most significant predictors of MACE. In addition, we provided an additional performance analysis on 3-point MACE (excluded revascularization) to better understand the predictivity of our fat-omics model on different outcomes.

Supplemental Figure 1 provided the process of our data exclusion, while Supplemental Table 1 showed characteristics of our 400-patient cohort for train/test subset. Supplemental Table 2 expanded on the predictive power of EAT volume from different spherical regions (SR), with special emphasis on the outermost layer (SR1), which demonstrated the highest significance in MACE prediction. This aligned with the anatomical fact that SR2 are proximal to the coronary arteries, also subjecting to pericoronary adipose analysis. Supplemental Table 3 delved into the EAT HU normalized distributions, examining the probability of voxel volumes within specific HU ranges. The highest bin (Pro_50_30) still showed high significance among all bins. Supplemental Table 4 presented analysis of EAT fixed thickness bins, offering insights into the distribution of thickness across predefined bins and their relationship to MACE, thus revealing the importance of EAT thickness heterogeneity in cardiovascular risk assessment. Supplemental Table 5 consisted of all selected EAT features by cox elastic net, underscoring the intricate relationship between EAT characteristics and cardiovascular health. All high-risk features mentioned in the main text were included in final fat-omics model. Supplemental Table 6 showed the corresponding coefficient of the same EAT features used in the final fat-omics model. In Supplemental Table 7, we performed additional performance analysis on 3-point MACE (excluded revascularization), while the final fat-omics model still outperformed all other accessed models with the highest testing C-index, 2-year AUC and AIC.


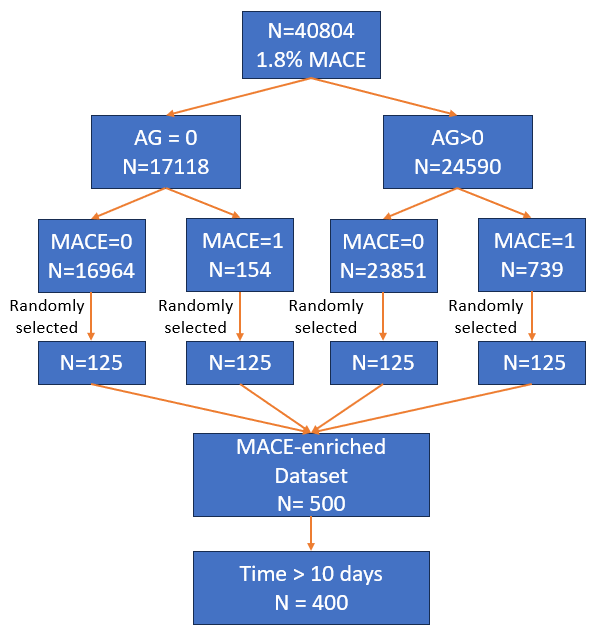


**Supplemental Figure 1:** Flow chart for data selection. Initially, the UH-clarify study comprised 40,804 patients (1.8% MACE rate). For this special study cohort, we identified 400 patients for whom we performed validated manual editing of EAT segmentation, which a time-consuming task (~1 hours per CTCS scan, done by trained analysts), to ensure the accuracy required for our fat-omics analysis. The selection process of those 400 cases involved matching patients based on balanced zero non-zero Agatston score with balanced MACE, no MACE cases.

**Supplemental Table 1:** Characteristics of the 400-patient cohort for train/test subset. There is no significant different between train/test cohort, indicating a fair train/test split.


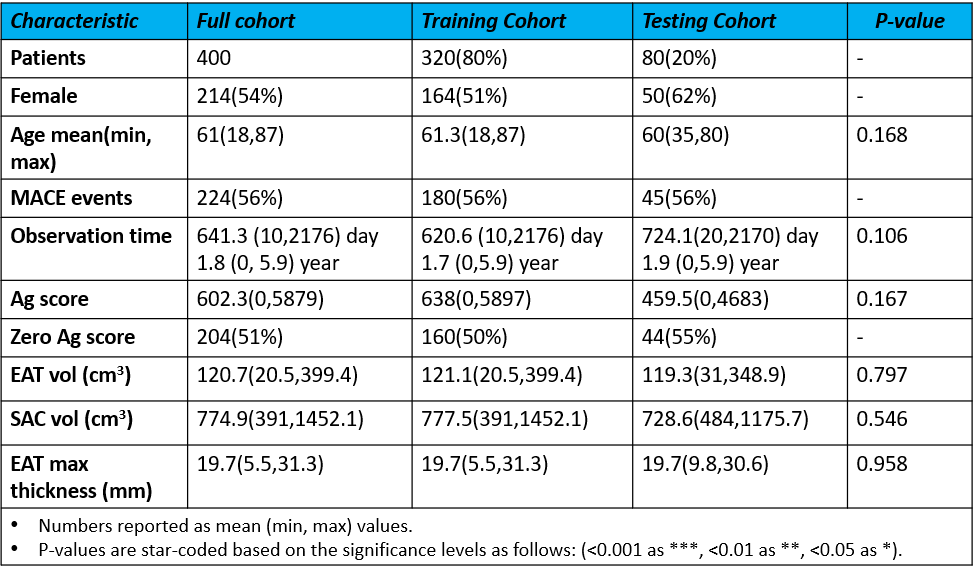


**Supplemental Table 2.** Univariable Cox modeling of EAT volumes, including the contributions from different shells sub-regions. Each row presents a univariable Cox proportional hazards model for the respective feature, including the hazard ratio, AIC, and corresponding p-value. The total EAT volume was divided into four quartiles shells from outer to inner (SR1-SR4), where the Vol_SR1 represents EAT volume at the most outer shell. Similar as slabs sub-regions analyses in Table 3, EAT in the outermost shell of the heart is the most significant with a C-index of 0.56. This aligns with the anatomical fact that SR1 is proximal to the coronary arteries, also subjecting to pericoronary adipose analysis.

**
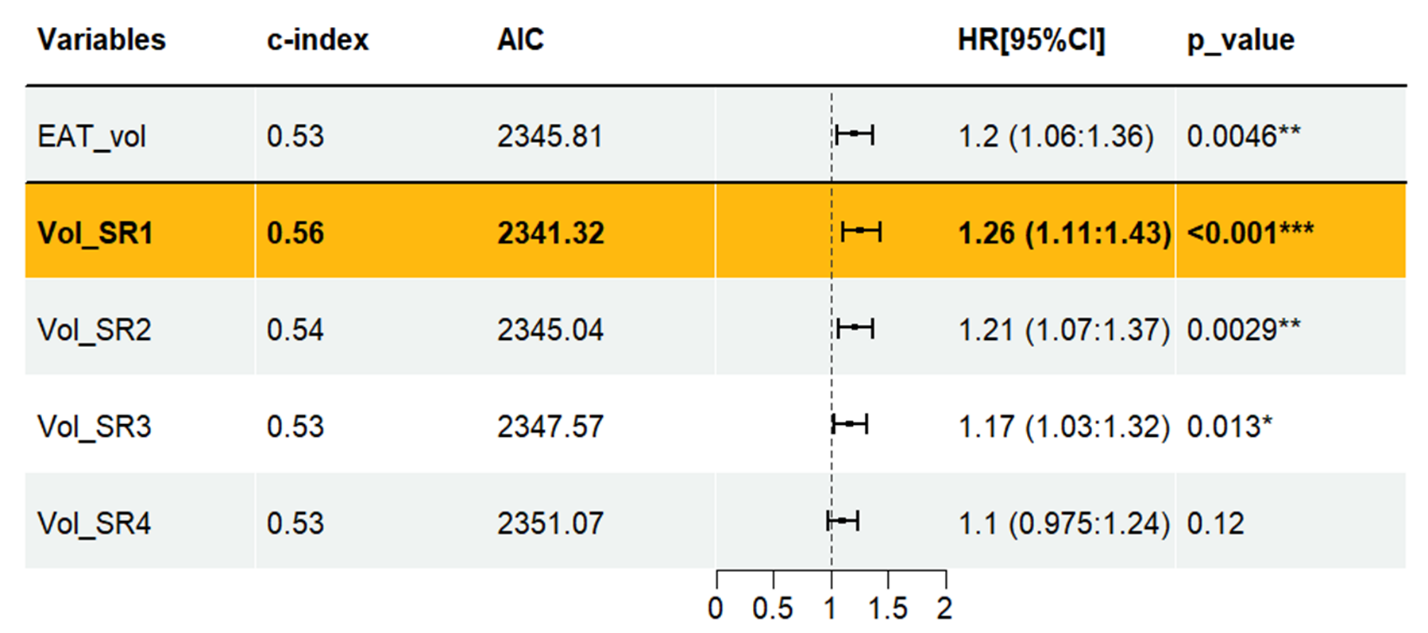
**

**Supplemental Table 3:** Univariable Cox analysis of EAT HU normalized distribution. In addition to just using the volumes of tissue within each range, we normalized histograms and obtained a probability of being within a range of HU values. Probability of HU range is analyzed (e.g., Pro_190_170 corresponds to the Probability of EAT having HU values between -190 and -170 HU). Results were only a little poorer than obtained in Table 4, while the highest bin still showed significance.

**
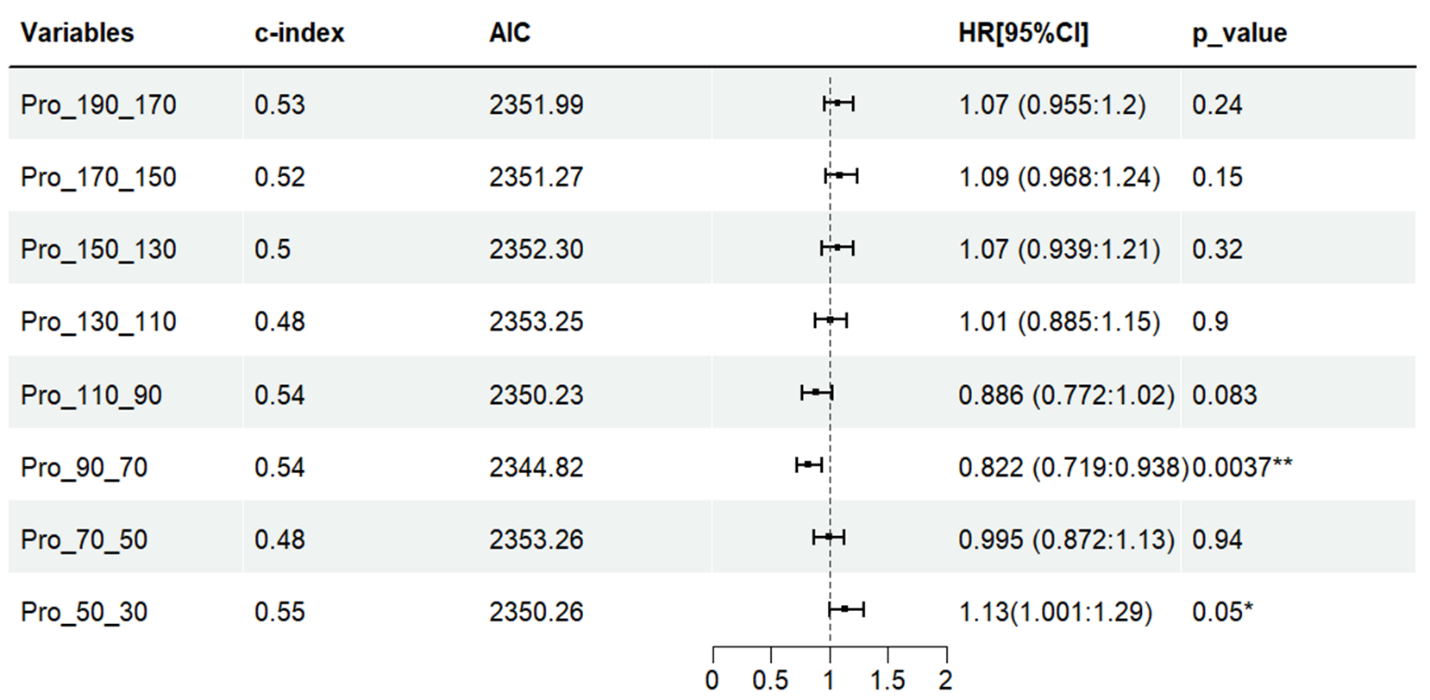
**

**Supplemental Table 4**: Fixed histogram bin analysis of EAT thickness. Similar to HU, we delved deeper by dividing the thickness measurements into four fixed histogram bins (each 8mm wide), determined by the spread of thickness observed across all patients. Probabilities of thickness are analyzed where thickness_bin44_Pro indicating the probability of thickness between 24 to 32mm. As expected, the largest thickness bin is the most significant feature, consistent with our findings in Fig. 4 (long tail thickness outlier is more significant).


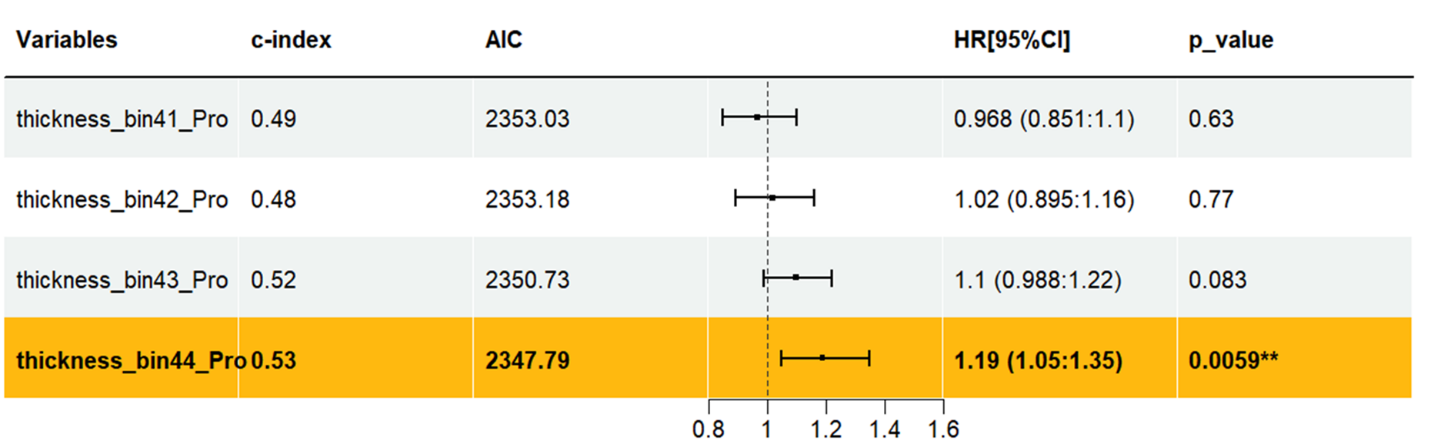


**Supplemental Table 5**: List of all selected EAT features in fat-omics. We started with 148 EAT features and performed maximum relevance minimum redundancy (MRMR) to exclude highly correlated features, resulting 50 uncorrelated EAT features. Among all pre-selected features, 15 dominant features were selected by cox elastic net. We performed cox proportional hazard analysis on those 15 selected features. Corresponding hazard ratio and p-value were listed in the table. All significant features introduced previously were included in this table.


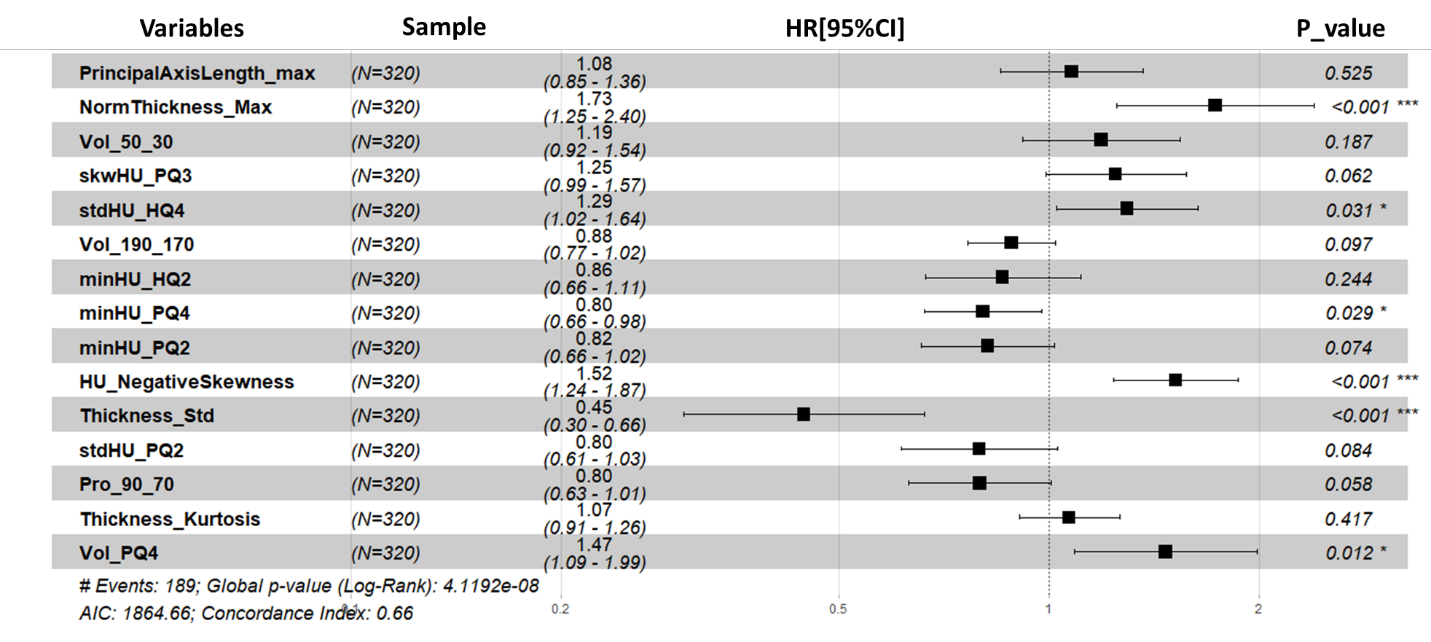


**Supplemental Table 6:** List of all 15 selected EAT features and corresponding coefficients in the multivariate fat-omics model.


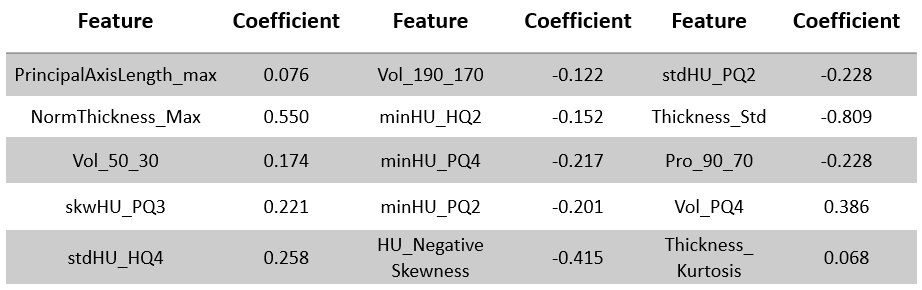


**Supplemental Table 7:** Risk prediction from our aggregated fat-omics model as compared to predictions from subsets of features for 3-point MACE (exclude revascularization). We used the same set of 15 features as 4-point MACE (corresponding coefficients were fine-tuned based on training set). Similar performance was observed. The fat-omics model still surpassed all other assessed models with the highest C-index, 2-year AUC, and lowest AIC for both training and held-out testing set.


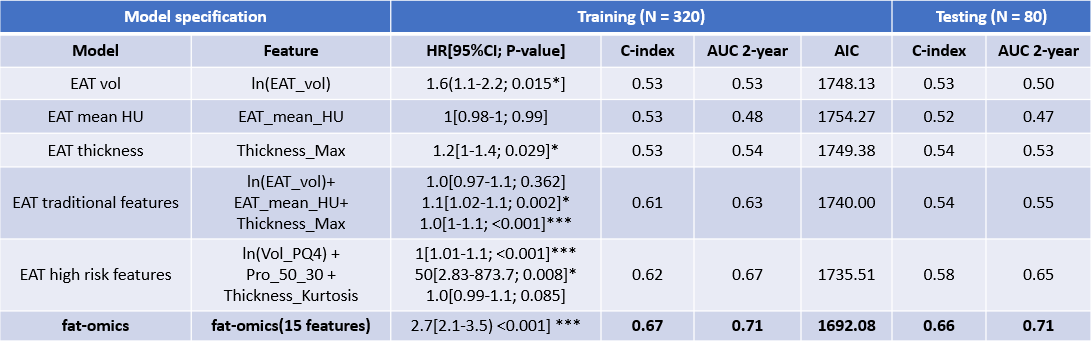

Supplement: Supplemental Material [file mmc1.docx]
